# Association or causation: still more questions than answers in early-onset gastrointestinal cancers

**DOI:** 10.1016/j.esmogo.2023.08.005

**Published:** 2023-11-07

**Authors:** E. Fontana, I. Ben-Aharon

**Affiliations:** 1 Sarah Cannon Research Institute UK, London, UK; 2 Division of Oncology, Rambam Health Care Center, Haifa; 3 Rappaport Faculty of Medicine, Technion, Haifa, Israel

The incidence of early-onset (EO) cancers (diagnosed before 50 years of age) of the gastrointestinal (GI) tract is on the rise.^1^ Trends were firstly observed in colorectal cancer (CRC), where the higher overall disease burden made changes more noticeable compared to less frequently diagnosed GI cancers. With close examination, stomach, pancreatic and hepatobiliary cancers have also appeared to increase. Trends are significantly different across countries, implying the presence of heterogeneous contributing elements for certain populations and protective ones for others. Incidence, prevalence and mortality data are not necessarily collected in the same standardised way globally, leaving gaps that are difficult to interpret, especially in the presence of low numbers with consequent limited power calculation.

Recent figures confirmed the continuous increase in the incidence rate of EO-CRC, from 4.2/100 000 in 1990 to 6.7/100 000 globally in 2019, with faster uptrends in EO-CRC (+1.6%) than in older patients (+0.6%). Trends significantly differ by region and country with East Asia and Jamaica among those with the fastest cancer burden increase.^2^

Several factors have been associated with EO-CRC, including the introduction of a Western diet, sedentary lifestyle, metabolic syndromes, obesity and diabetes, use of antibiotics with consequent dysbiosis and chronic mucosal inflammation.^1^ Often though, contradictory results can be found in the literature due to the complexity of meta-analyses, biases from retrospective studies, differences in study control groups assessed for comparisons (in some studies healthy young individuals, in some others average-onset CRC patients) and frequent overrepresentation of a North American population.

Furthermore, risk factors are not always evident; often young, so-far healthy individuals with no significant medical history present to our clinics lacking any obvious cause. This serves to remind us that association does not imply causation.^3^ While the bulk of evidence for recognised risk factors continues to rise together with problem awareness, determining causality explaining the rising incidence and understanding why this is observed in certain countries but not in others remains the next challenge. Inflammation is the most plausible causal suspect of the rising incidence of EO-CRC; however, what triggers the inflammation (in particular in the presence of exogenic exposure to pollutants, specific drugs or certain diets), when the process starts (whether during adolescence or early adulthood) and how long an exposure is required to initiate cancer on an individual basis seem still far from our understanding.

Recently, Park et al.^4^ described a significant association between non-alcoholic fatty liver disease (NAFLD) and increased risk of EO-GI cancers using data extracted from the South Korean Health Insurance Service (KNHIS) database over a 4-year period from 2009 to 2012. This includes anthropometric measures, laboratory tests, medical history and lifestyle data collected within the National Health Screening programme from insured individuals older than 20 years of age every 2 years. Nearly all South Koreans have insurance and up to 76% of eligible individuals participate in the screening programme, making the data well representative. The incidence of NAFLD was assessed using one of the best validated fatty liver indices and an established cut-off, previously validated in the general Korean population. Among 5 265 590 participants aged 20-39 years, NAFLD was present at baseline in 25.4% of the population. A total of 14 565 out of over 5 million participants (0.28%) developed gastrointestinal cancers by the end of 2018. The vast majority were diagnosed with CRC (*n* = 8673) followed by stomach (*n* = 5547), pancreatic (*n* = 2288), liver (*n* = 1299), biliary tract (*n* = 434), gallbladder (*n* = 147) and oesophageal (*n* = 88) cancer. These were individuals with a higher prevalence of high body mass index (BMI), blood pressure, fasting glucose levels and smoking habits compared to individuals who did not develop GI cancers. Individuals with high alcohol intake, liver cirrhosis and hepatitis, as well as participants with prior history of cancer or with a diagnosis of cancer during the first year of follow-up were excluded from the analysis to minimise the influence of pre-existing disease. The incidence of any other type of cancer was not described. The incidence rate of GI cancers per 100 000 person-years was higher in the presence of NAFLD than in the absence of NAFLD (*P* < 0.01). Of note, NAFLD showed a significant association with an increased risk of overall digestive tract cancers even after adjusting for confounders (age, sex, BMI, smoking status, alcohol consumption, physical activity, income status, diabetes, pancreatitis and cholangitis). The authors concluded that NAFLD is a modifiable factor that could be managed to reduce the risk of young-onset digestive cancer, although a causal and longitudinal relationship could not be established.

This is a nationwide cohort with over 5 million individuals including data prospectively collected from a well-established screening programme: these are the significant strengths of this study. Considering the nature of the KNHIS programme and the characteristics, lifestyle and risks factors of the Korean population, it is unclear how generalisable and reproducible these findings are in other countries. With an incidence of 12.9 cases/100 000 inhabitants and an average annual percent change (APC) of +4.2% between 2008 and 2012, EO-CRC incidence in South Korea has been the highest among 42 countries. Interestingly though, between 2011 and 2016 the incidence of EO-CRC significantly decreased in both men (−5.91 APC) and women (−7.73 APC), in the absence of any change in screening recommendations.^5^ Conversely, a systematic review on the prevalence of NAFLD in South Korea demonstrated a significant increase of NAFLD from 29% (2001-2007) to 31% (2008-2014), with a higher prevalence in obese individuals.^6^

Globally, NAFLD is estimated to be present in around 30% of adults, higher in males (40%) than in females (26%) and with significant variation across world regions.^7^ While in Asia there is a high prevalence of lean and non-obese NAFLD, in North America the high prevalence at 48% is driven by obesity, especially within Hispanics, where the patatin-like phospholipase domain-containing protein 3 (*PNPLA3*) mutation is associated with the inflammatory non-alcoholic steatohepatitis (NASH). In Europe, Italy ranks second for prevalence (38%) after Turkey (48%), while the lowest prevalence is observed in France (18%).^7^ Conversely, EO-CRC remains low with decreasing APC (−2%) in Italy, while France registered an increase of APC between 4% and 5%.^8^

Taking together, this evidence suggests that the association of NAFLD and EO-CRC might be different in different countries. Nevertheless, NAFLD is part of an inflammatory picture with systemic release of proinflammatory cytokines and alteration in the microbiome which could lead to carcinogenesis. Based on the current study,^4^ NAFLD is associated with EO digestive cancers in South Korea, where both conditions are at a relatively high prevalence; however, since the association, but so far not causation, has been established, reducing or controlling NAFLD might not necessarily result in a decreased incidence of cancer. The study lacks insight into the possible causes of NAFLD in young adults, whether mainly driven by metabolic syndrome or whether pharmaceutical exposure to medications (e.g. corticosteroids, oestrogens, antidepressants or antibiotics) may potentially also contribute to the pathogenesis of NAFLD and/or indirectly to the pathogenesis of EO-CRC in the population included.

Understanding the causes remains crucial to revert these increasing trends and prevent the development of new cases of EO-GI cancers; identifying high-risk individuals is a key element for early detection of new cancers and prevention of neoplastic transformation of pre-malignant lesions that can be endoscopically removed. Encouraging a healthy lifestyle, including physical activity, non-smoking habits, a balanced diet and weight control, is certainly recommended, but might not be enough to prevent cancer. The strongest known risk factor for CRC is having a first-degree relative (FDR) with a diagnosis of CRC. In this situation, screening colonoscopies are recommended to start at 40 years of age. Over one quarter of EO-CRC patients have an FDR with a history of CRC or adenomas. Unfortunately, figures from the United States show that <40% of high-risk individuals aged between 40 and 49 years have had a screening colonoscopy.^9^

The rising incidence of EO-CRC seems to be related to sporadic cases with no obvious differences in genomic drivers seen compared to older patients.^1^ However, germline cancer susceptibility gene alterations are present in up to 16% of EO-CRC, prompting the recommendation of germline multigene panel testing for all individuals with a personal history of CRC at age <50 years.^9^ This recommendation will hopefully have a positive impact in the near future in identifying germline cancer-related gene alterations in patients. This may ultimately improve cascade testing and screening interventions in family members through enhanced identification and prevention strategies for the related individuals at risk.

Importantly, not only cancer-related germline alterations are risk elements: germline variation in inflammation-related pathways, including cytokine signalling, oxidative stress and human leukocyte antigen amongst others, have been related to oesophageal adenocarcinoma in a genome-wide association study.^10^ These germline alterations remain widely unexplored by standard routine multigene panel testing.

Therefore, genetic susceptibility for both cancer and inflammation pathways as pre-existing risk factors, their interaction with environmental exposure and host-related influences in different populations remain a research priority. Educating health care providers and the wider population about the most up-to-date recommendations for screening and germline testing is essential and requires fast and effective implementation.

## Funding

No funding to disclose.

## Disclosure

Given their role as a Editor-in-Chief, Irit Ben-Aharon and Associate Editor Elisa Fontana had no involvement in the peer review of this article and has no access to information regarding its peer review. Full responsibility for the editorial process for this article was delegated to Florian Lordick, Editor-in-Chief, of the Journal.

## References

[1] Ben-Aharon I., van Laarhoven H.W., Fontana E., Obermannova R., Nilsson M., Lordick F. (2023). Early-onset cancer in the gastrointestinal tract is on the rise—evidence and implications. Cancer Discov.

[2] Wang Y, Huang X, Cheryala M, et al. Global increase of colorectal cancer in young adults over the last 30 years: an analysis of the Global Burden of Disease Study 2019. *J Gastroenterol Hepatol*. 2023. 10.1111/jgh.16220 37211529

[3] Altman N., Krzywinski M. (2015). Points of significance: association, correlation and causation. Nat Methods.

[4] Park J.H., Hong J.Y., Shen J.J. (2023). Increased risk of young-onset digestive tract cancers among young adults age 20-39 years with nonalcoholic fatty liver disease: a Nationwide Cohort Study. J Clin Oncol.

[5] Shin A., Jung K.W., Jeong S.Y. (2023). Right then, wrong now: early-onset colorectal cancer in Korea. Cancer Res Treat.

[6] Im H.J., Ahn Y.C., Wang J.H., Lee M.M., Son C.G. (2021). Systematic review on the prevalence of nonalcoholic fatty liver disease in South Korea. Clin Res Hepatol Gastroenterol.

[7] Teng M.L., Ng C.H., Huang D.Q. (2023). Global incidence and prevalence of nonalcoholic fatty liver disease. Clin Mol Hepatol.

[8] Vuik F.E., Nieuwenburg S.A., Bardou M. (2019). Increasing incidence of colorectal cancer in young adults in Europe over the last 25 years. Gut.

[9] Cavestro G.M., Mannucci A., Balaguer F. (2022). Delphi initiative for early-onset colorectal cancer (DIRECt). International Management Guidelines. Clin Gastroenterol Hepatol.

[10] Buas M.F., He Q., Johnson L.G. (2017). Germline variation in inflammation-related pathways and risk of Barrett’s oesophagus and oesophageal adenocarcinoma. Gut.

